# 

**Published:** 1868-02

**Authors:** 


VOL. XXII. No. 2.

### THE
### Dental Register.

EDITED BY

J. TAFT & G. WATT.

FEBRUARY, 1868.

MONTHLY:
THREE DOLLARS PER ANNUM, IN ADVANCE.

CINCINNATI:
WRIGHTSON & CO., Printers, 167 WALNUT STREET.

*J. & WM. TAFT, Dentists, 238 West Fourth St.*

